# Deciphering Novel Antimicrobial Peptides from the Transcriptome of *Papilio xuthus*

**DOI:** 10.3390/insects11110776

**Published:** 2020-11-10

**Authors:** Joon Ha Lee, Hoyong Chung, Yong Pyo Shin, Mi-Ae Kim, Sathishkumar Natarajan, Karpagam Veerappan, Seong Hyun Kim, Junhyung Park, Jae Sam Hwang

**Affiliations:** 1 Department of Agricultural Biology, National Institute of Agricultural Sciences, 166, Nongsaengmyeong-ro, Iseo-myeon, Wanju-gun, Jeollabuk-do 55365, Korea; coover@korea.kr (J.H.L.); shinyp2020@korea.kr (Y.P.S.); kimma@korea.kr (M.-A.K.); ichibbang@korea.kr (S.H.K.); 2 3BIGS CO. LTD., 156, Gwanggyo-ro, Yeongtong-gu, Suwon-si, Gyeonggi-do 16506, Korea; hychung@3bigs.com (H.C.); sathish@3bigs.com (S.N.); karpagam@3bigs.com (K.V.)

**Keywords:** *Papilio xuthus*, bacterial infection, fungal infection, de novo transcriptome assembly, antimicrobial peptides, papiliocin

## Abstract

**Simple Summary:**

Insects live in a pathogenic microorganism rich environment. Thus, insects develop a stronger defense in terms of innate immunity. Antimicrobial peptides (AMPs) are one of the key tools in the insect’s innate immune system, which kills the invading pathogens. In this study, we used *Papilio xuthus*, the Asian swallowtail butterfly, to identify the AMPs from their genomic product. We utilized next generation sequencing technology and in silico analysis tools to decipher the possible novel AMPs. The obtained novel AMPs were then tested for the antibacterial and antifungal activities. Seven novel peptides were identified as the antimicrobial agent, and these can be used as a lead candidate in the process of antibacterial therapy development against various infectious diseases.

**Abstract:**

An insect’s innate immune system is the front line of defense against many invading microorganisms. One of the important components of this defense system is antimicrobial peptides (AMPs). Papiliocin is a well-studied antimicrobial peptide (AMP) isolated from the swallowtail butterfly, *Papilio xuthus*, and it was previously reported to be effective against Gram-positive bacteria, Gram-negative bacteria, and fungi, particularly in drug resistant Gram-negative bacteria. Hence, we aimed to identify novel AMPs from *Papilio xuthus* using its transcriptome. We immunized the swallowtail butterfly with *Escherichia coli, Staphylococcus aureus, Candida albicans*, and the total RNA was isolated. De novo transcriptome assembly and functional annotations were conducted, and AMPs were predicted using an in-silico pipeline. The obtained 344,804,442 raw reads were then pre-processed to retrieve 312,509,806 (90.6%) total clean reads. A total of 38,272 unigenes were assembled with the average length of 1010 bp. Differential gene expression analysis identified 584 and 1409 upregulated and downregulated genes, respectively. The physicochemical, aggregation, and allergen propensity were used as filtration criteria. A total of 248 peptides were predicted using our in-house pipeline and the known AMPs were removed, resulting in 193 novel peptides. Finally, seven peptides were tested in vitro and three peptides (Px 5, 6, and 7) showed stronger antimicrobial activity against Gram-negative bacteria and yeast. All the tested peptides were non-allergens. The identified novel AMPs may serve as potential candidates for future antimicrobial studies.

## 1. Introduction

The innate immune system is the front line of defense against an invading microorganism in all living organisms, especially insects. One of the important components of this defense system is antimicrobial peptides (AMPs). AMPs are effective against Gram-positive bacteria, Gram-negative bacteria, fungi, viruses, and parasites. The first insect AMP (cecropin) was isolated and studied in 1980s and since then, 318 insect AMPs have been cataloged to date in the Antimicrobial Peptide Database [1]. Based on the sequence and structure, insect AMPs are broadly classified into three groups: peptides with α-helices (e.g., Cecropins), β-sheet peptides with conserved cysteine residues (e.g., Defensins), and linear proline or glycine rich peptides (e.g., Drosocin, Attacins) [2,3,4,5]. Although insect AMPs are diversified in sequence and structural features, they share some common functionality such as a net positive charge and inclusion of approximately 50% hydrophobic residues. These properties contribute majorly to their bactericidal and antifungal activity. These features can be a key factor in the screening and development of AMP-based antimicrobial therapeutics [6].

*Papilio xuthus (P. xuthus)*, commonly known as the swallowtail butterfly, is found in the far east Asian regions of Korea, Japan, China, Taiwan, and Myanmar. Papiliocin is the 37 residue α-helical cecropin-like AMP isolated from an immune challenged larvae of swallowtail butterfly [7]. Papiliocin has exhibited the bactericidal activity against both Gram-positive and Gram-negative bacteria, but the effect is more pronounced in Gram-negative strains. Additionally, the antibacterial and antifungal activity of papiliocin is observed in multidrug- resistant bacterial and fungal strains, respectively. Also, it is reported to possess an anti-inflammatory activity and non-toxicity toward mammalian cells [8,9,10]. These reported properties of papiliocin renders it as a potential antimicrobial from swallowtail butterfly that can be used as an alternative or a supportive for classical antibiotics. AMPs may serve as an effective antimicrobial against antimicrobial resistance, a growing concern in clinical settings.

In this study we have aimed to discover novel AMPs from the swallowtail butterfly. In this context, we utilized the next generation sequencing (NGS) technology in deciphering the transcriptome of immunized *P. xuthus.* We used our AMP prediction pipeline, a step-by-step screening of physiochemical activity, which also included allergen testing. After the initial screening, the peptides were blasted with protein databases and the known AMPs were removed. Finally, the screened peptides were evaluated for the antimicrobial activity and we found seven novel AMPs from *P. xuthus*.

## 2. Materials and Methods

### 2.1. Microorganisms and Growth Conditions

The bacterial strains *Escherichia coli* (KACC 13821, ATCC 11775) and *Staphylococcus aureus* (KACC 10768, ATCC 25923), and the yeast strain *Candida albicans* (KCTC 7121, ATCC 14053) were purchased from the Korean Agricultural Culture Collection (KACC) and Korean Collection for Type Cultures (KCTC). Both the bacteria and yeast were grown overnight in tryptic soy broth (TSB; Difco, Thermo Fisher Scientific, Waltham, MA, USA) at 37 °C, 200 rpm to the stationary phase. Bacteria were re-inoculated in fresh TSB medium and cultivated for 3 h to the log phase. The strains were stored with 15% glycerol at −70 °C.

### 2.2. Insect and Immunization

*Papilio xuthus* were reared at room temperature with 60% ± 5% relative humidity and a 16/8 light/dark photoperiod cycle. For immunization, each last instar larva was injected with the mixture of *Escherichia coli*, *Staphylococcus aureus*, and *Candida albicans* (5 × 10^3^ colony-forming units) suspended in 5 μL sodium phosphate buffer (10 mM; pH 7.4). For the non-immunized control, each larva of swallowtail butterfly was injected with the 5 μL sodium phosphate buffer alone (10 mM; pH 7.4). After maintaining both the immunized and non-immunized larvae at 25 °C ± 1 °C for 18 h, total RNA was isolated.

### 2.3. Transcriptome Sequencing, De Novo Assembly, and Functional Annotations

Briefly, total RNA from three non-immunized and three immunized larvae were isolated for whole transcriptome sequencing. For the total RNA isolation, the larvae were washed with PBS and sterilized with 70% ethanol and then anesthetized on ice. RNA was isolated from the whole body using TRIZOL reagent (Invitrogen, Carlsbad, CA, USA) according to the manufacturer’s instructions. The total RNA quality and quantity of each sample were analyzed by a Bioanalyzer 2100 system using an RNA 6000 Nano Kit (Agilent technologies, Inc., Santa Clara, CA, USA). Approximately 2 μg of total RNA ((RNA Integrity Number (RIN) >7.0) of each sample was used to construct transcriptome cDNA libraries using a Truseq Stranded mRNA Prep Kit (Illumina Technologies, San Diego, CA, USA), according to the manufacturer’s instructions. Further, cDNA libraries were sequenced using an Illumina HiSeq 2500 platform to generate 100 bp paired end reads. An initial raw data quality was checked by the FastQC (https://www.bioinformatics.babraham.ac.uk/projects/fastqc/). Then, Trimmomatic [11] was used to remove low quality reads and adaptor contents using default parameters. Further, the Trinity program [12] was used for the de novo assembly of high-quality reads with default parameters. The redundancy transcripts were identified through Cluster Database at High Identity with Tolerance CD-HIT-EST [13] with an identify threshold of 95% sequence similarity. In addition, TransDecoder [12] was used to identify the longest ORF per transcript and protein fragment for the peptide prediction. Furthermore, functional annotations of the filtered unigenes were identified through a homology search against the Swiss-Prot databases by BLASTX with an E-value cut-off of 10^−5^. Meanwhile, domains were identified by using InterProScan [14]. The assembled unigenes were subjected to a GO enrichment analysis by the GOseq Bioconductor package [15] based on the Wallenius non-central hypergeometric distribution with the corrected *p*-value less than 0.05.

### 2.4. Expression Analysis

To identify the differentially expressed genes (DEG), initially cleaned raw reads were mapped to the assembled unigenes with Bowtie [16] using default parameters. The gene transcript abundance was calculated as transcripts per kilobase million (TPM) [17] using the RNA-Seq by Expectation-Maximization (RSEM) package [18]. The generated read counts of all genes were given as input for the identification of differential expressed genes (DEG) using EdgeR [19]. The cut off was set as *p*-value  < 0.05 and log2 Fold Change (log2FC) ≥ 1 for the downstream analysis.

### 2.5. Antimicrobial Peptide (AMP) Prediction and Classification

A modified in silico strategy was employed to predict AMPs from the deduced amino acid sequences. Briefly, peptides were screened for physiochemical propensity, followed by mapping and prediction in the peptide database and CAMP classifier, respectively. The in silico pipelines were run with parameters defined previously [20] to predict the physiochemical property. The AMPs were then mapped with the CAMP database [21] and classified as novel or known AMPs. Two programs, PatMatch (no mismatch) for sequences ≤20 residues in length [22] and BLASTP (1E-05) for sequences ≥20 residues in length were used. The BLAST results were filtered with a similarity score ≥90. Sequences with the observed similarity at the given cutoff values were considered as known AMPs, and the others were considered as novel AMPs. Finally, the novel AMPs were manually validated for continuous stretches of amino acids to account for the low-complexity regions and assembly artifacts.

### 2.6. Peptide Synthesis

All the novel peptides selected based on the various prediction tools above-mentioned [23] were synthesized using solid-phase peptide synthesis methods at AnyGen Co. Ltd. (Gwangju, Korea). Then, each peptide was purified to >95% by high-performance liquid chromatography, and the purity was confirmed by mass spectrometry analysis. The peptides were dissolved in acidified distilled water (0.01% acetic acid) and stored at −20 °C until used in subsequent experiments.

### 2.7. Antimicrobial Activity Assay

The radial diffusion assay was performed to test the antimicrobial activities of peptides, as described previously with slight modifications [24]. In brief, bacteria and yeast strains were grown to the mid-logarithmic phase in TSB at 37 °C and then washed twice with 10 mM sodium phosphate buffer (pH 7.4). A total of 4 × 10^6^ CFU was added to 10 mL of an underlay agarose gel [9 mM sodium phosphate, 1 mM sodium citrate, pH 7.4, 1% (*w/v*) Type I (low electroendosmosis) agarose (Sigma, St. Louis, MO, USA), and 3% TSB (Difco, USA)]. The underlay gel was poured into a 100-mm INTEGRIDTM Petri dish. After the agarose solidification, 3-mm-diameter wells were punched and 5 μL of each peptide solution was added to each well. Buffer alone was used as a negative control. Plates were incubated at 37 °C for 3 h to allow for diffusion of the peptides. The underlay gel was then covered with the 10 mL of nutrient-rich agar overlay (6% TSB and 1% agarose). The antimicrobial activity of a peptide was measured as the diameter of the cleared zone around each well after 12 h of incubation at 37 °C. This experiment was repeated at least three times independently.

### 2.8. Hemolytic Assay

This experiment was approved by the Institutional Animal Care and Use Committee (IACUC) of the National Institute of Agricultural Sciences (approval number: NAS-202014). The hemolytic activity of the peptides was determined by monitoring the release of hemoglobin from mouse erythrocytes at 540 nm. For the hemolytic assay, 20 μL of each peptide solution at a predetermined concentration was added to 180 μL of a 2.5% (*v/v*) suspension of mouse erythrocytes in phosphate-buffered saline (PBS). Melittin (Sigma, USA), a hemolytic and α-helical peptide isolated from bee venom, was used as the positive control. This mixture was incubated for 30 min at 37 °C, and 600 μL of PBS was then added to each tube. After 3 min of centrifugation at 10,000× *g*, the supernatant was removed, and the absorbance was measured at 540 nm. Evaluations were made from the results of at least three independent experiments, each carried out in triplicate.

### 2.9. Data Availability

All the raw data utilized in this study were deposited at the National Center for Biotechnology (NCBI) Sequence Read Archive (SRA) under the accession number SRA1096681.

## 3. Results and Discussion

### 3.1. Illumina Sequencing and De Novo Transcriptome Assembly

**Figure 1:**
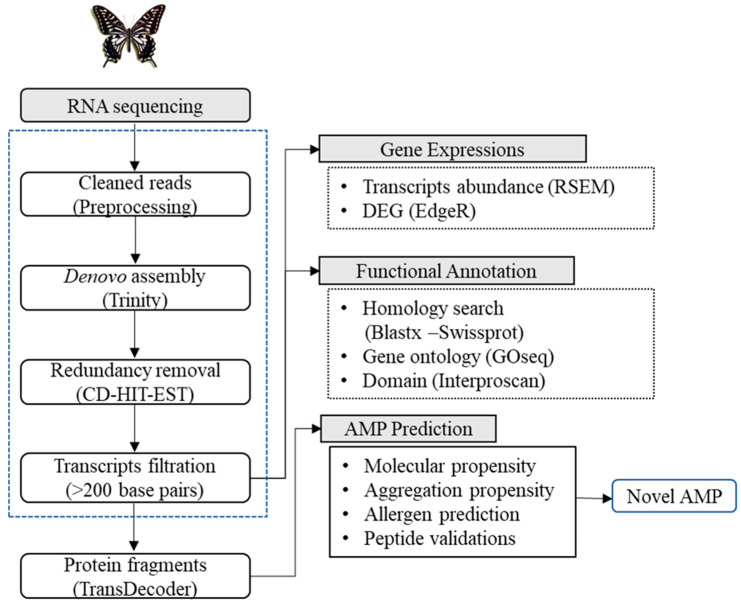
Overall workflow of de novo transcriptome assembly and antimicrobial peptide (AMP) prediction of *Papilio xuthus.*

Three biological replicates for each group, a total of six samples (non-immunized vs. immunized) were sequenced and the total number of raw reads obtained in each group is summarized in Table S1. Overall workflow of *P. xuthus* de novo transcriptome assembly and the AMP prediction are illustrated in Figure 1. First, adapter and low-quality sequences were removed, and the resultant total clean reads were 90.6% (312,509,806) and the individual clean reads ranged between 89.1% and 93.48%. (Table S1). An Illumina quality control score of Q30 (incorrect base call accuracy was 1 in 1000) was observed to be high in all sequenced samples, which was indicative of good quality data (Table S1). The obtained clean reads were then assembled de novo using “Trinity”, a software package for transcriptome assembly. A total of 38,272 unigenes were assembled with the average length of 1010 bp. Unigenes with the length varying from 200 to >2000 bp were obtained and the unigene with >200 bp length was considered for further processing.

### 3.2. Functional Annotations and Differential Gene Expression Analysis

**Figure 2:**
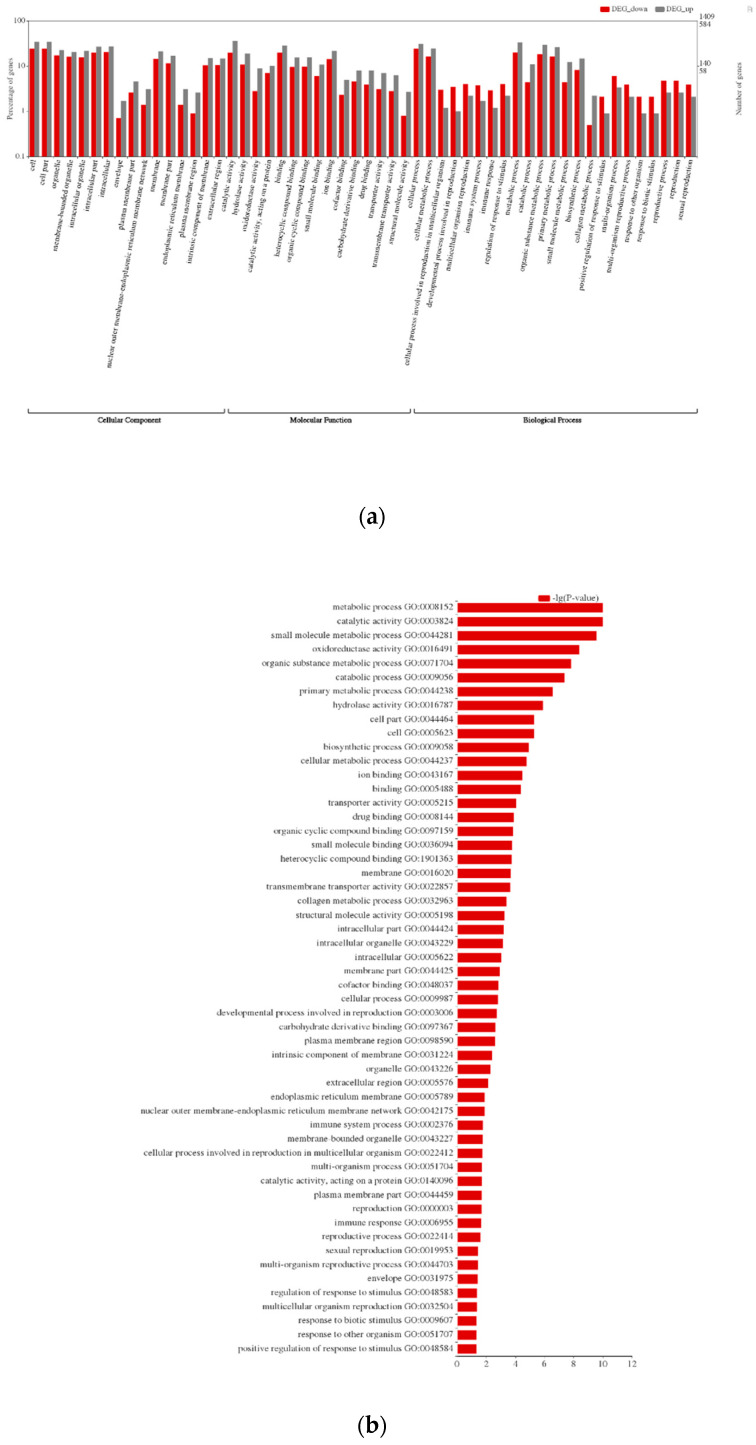
Gene ontology (GO) classification of the *Papilio xuthus* transcriptome. (**a**) The GO was summarized into three main categories: biological process, cellular component, and molecular function. *x*-axis indicates the sub-categories of GO terms, and the *y*-axis indicates percentage and number of unigenes on the left and right side, respectively. (**b**) Top fitting GO subcategories were displayed.

The databases Swiss-Prot, GO, and InterProscan were used to annotate the assembled data. A total of 19,900 (52%) unigenes were annotated by the BLASTX search against the Swiss-Prot and about 13,842 (36%) unigenes were annotated using InterProscan (Table S1). The gene function prediction by GO analysis resulted in 13,945 annotated genes distributed in three major categories, biological process, cellular component, and molecular function (Figure 2a). To induce the immune system, we immunized *P. xuthus* with the mixture of Gram-negative, Gram-positive bacteria, and fungus. DEG analysis was carried out to find the differential expression pattern of the immunized and non-immunized *P. xuthus*. We obtained a total of 1993 differentially expressed transcripts based on the cutoff log2FC ≥ 1 and *p*-value < 0.05. Among these, 584 transcripts were upregulated, and 1409 transcripts were downregulated in the immunized group when compared with the control. The DEGs were subjected to GO enrichment analysis and the top five subcategories were metabolic process, catalytic activity, small molecule metabolic process, oxidoreductase activity, and organic substance metabolic process (Figure 2b).

### 3.3. AMP Prediction

AMP prediction from the insect transcriptome using the computational approach is our long-standing idea from which we have continuously thrived in testing [20,25,26]. The natural environment of insect livelihood is rich in harmful bacteria, fungus, parasites, and viruses, thus there is a need for an efficient defense system [3]. Several AMPs identified from insects have been reported to possess antibacterial, antifungal, and antiviral activities that can be utilized for the therapeutic design of antimicrobials. Papiliocin is an AMP with antimicrobial and anti-inflammatory activities and was previously isolated from *P. xuthus* [10]. To retrieve a biological insight of the other promising AMPs from swallowtail butterfly, we immunized and screened its transcriptome using a state of art in silico pipeline (Figure 1). Continuing expansion of NGS technology and related data analysis was advantageously utilized in understanding the immune related genes or peptides in the insects [27,28]. *P. xuthus* AMP prediction involves several physiochemical measures such as residue length, charge, aggregation propensity, and allergen prediction. AMPs’ modes of action primarily involve membrane destabilization for which the positively charged AMP is critical for binding with the negatively charged membrane components. Thus, the bactericidal or antifungal activities of AMPs depend on its physiochemical properties such as length, isoelectric point (pI), and charge. In the case of aggregation property, the AMPs are desired not to possess in vitro aggregation because it decreases its activity and availability. However, in vivo aggregation on bacterial cell surfaces helps in bacterial clearance from the host. Additionally, AMP containing the protease cleavage site was removed to avoid degradation in the human body. The allergen prediction parameter was also included in this study to avoid the extended usage of AMP in in-vitro and clinical settings. All the predicted AMPs were non-allergen. Collectively, all the peptides were passed through the step-by-step physiochemical filtration schema. The detailed cutoff values are mentioned in the Materials and Methods Section 2.5 and Table S2. The peptides that pass all the physiochemical propensity were then mapped to the CAMP database to identify novel AMPs. In total, 248 AMPs passed through all filters were predicted to be novel putative AMP.

### 3.4. Experimental Validation of Putative and Novel AMPs

**Figure 3:**
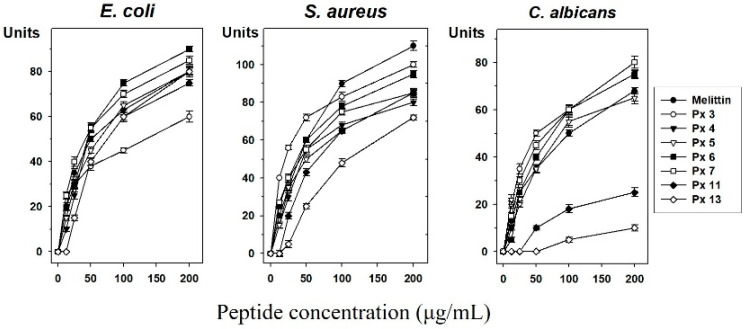
Antimicrobial activity assay. Antimicrobial activities of seven selected peptides against *Escherichia* *coli, Staphylococcus aureus*, and *Candida albicans* determined by a radial diffusion assay. Peptide concentration (*x*-axis) was plotted against the diameter of the microbial growth inhibition zone (*y*-axis) after incubation for 12 h and is expressed in units (1 mm = 10 units). Melittin was used as a positive control. Mean values were obtained from tests repeated three times.

After the in silico prediction, 193 novel AMPs with potentially high activity were ultimately selected. Among them, 14 peptides were synthesized according to the results of the secondary structure prediction [29]. We chose the α-helix regions based on secondary structure due to the efficiency and cost of peptide synthesis for the development of antimicrobial agents. After an initial screening of the synthesized peptide, we finally selected seven AMPs (Table 1 and Table 2) and tested their antimicrobial activities against *E. coli*, *S. aureus*, and *C. albicans* using a radial diffusion assay (Figure 3). We found antimicrobial activity in seven synthetic peptides (Px 3, 4, 5, 6, 7, 11, 13), which increased in a dose-dependent manner. Melittin was used as a positive control. Remarkably, the peptides Px 4, 6, 7 showed stronger antimicrobial activity than melittin and Px 5, 13 showed similar antimicrobial activity with melittin in *E. coli*. The antimicrobial activities of Px 3 showed similar antimicrobial activity with melittin in *S. aureus*. Correspondingly, Px 3, 6, 7 showed stronger antimicrobial activity than melittin and Px 4, 5 showed similar antimicrobial activity with melittin against *C. albicans*. Altogether, the results showed that Px 5, 6, and 7 had a broad range of activities toward Gram-negative bacteria and yeast.

**Figure 4:**
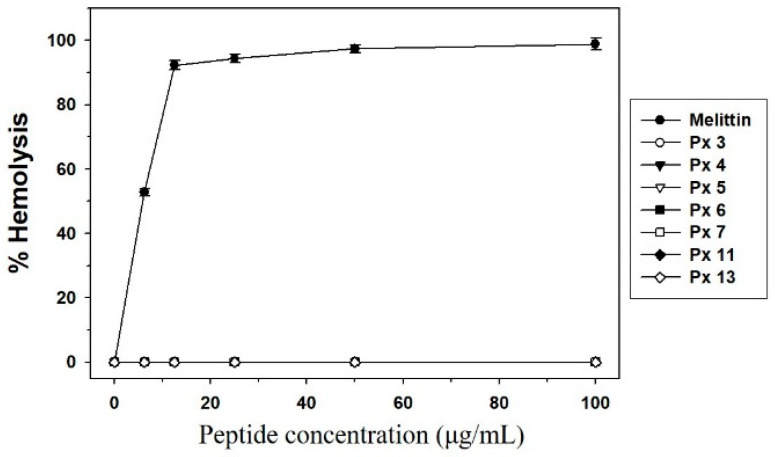
Hemolytic activity of the seven selected peptides. Peptide concentration (*x*-axis) was plotted against the percentage of hemolysis (*y*-axis) of mouse red blood cells after incubation for 30 min. Melittin was used as the positive control. The percent hemolysis was calculated with the following equation: hemolysis (%) = (A_540_ of sample − A_540_ of peptide-free control)/(A_540_ of 100% control − A_540_ of peptide-free control) × 100. Each symbol represents the mean value estimated from triplicate experiments.

The seven selected synthetic peptides showing antimicrobial activity in the radial diffusion assay were studied for hemolysis effect (Figure 4). Melittin lysed 92% of mouse red blood cells at a concentration of 12.5 μg/mL, whereas no hemolytic activity was observed for the seven synthetic peptides at this concentration and up to 100 μg/mL. Melittin has strong and broad antimicrobial spectrum, but the peptide lacks selectivity toward microbes. The purpose of this experimental study was to find novel peptides that have potent antimicrobial activities with little or no cytotoxicity. Thus, these data indicate that the selected peptides are useful for the development of novel antimicrobial agents.

## 4. Conclusions

In this study, we aimed at revealing novel AMPs from the immunized *P. xuthus* transcriptome. We obtained seven peptides from in silico screening, which were also tested in vitro for the antimicrobial activity and toxicity toward normal cells. Our work will be a resource for both the transcriptome data and AMP discovery in swallowtail butterfly.

## Figures and Tables

**Table 1:** List of the selected seven peptides.

Peptide	Sequence	Length	Mw (Da)
Px 3	R to L *	15	1797.3
Px 4	V to S *	18	2213.6
Px 5	Y to L *	17	2216.7
Px 6	H to K *	16	1838.3
Px 7	R to Y *	20	2501.1
Px 11	F to R *	15	1878.2
Px 13	S to K *	14	1685.1

* signify C-terminal amidation. Mw, molecular weight.

**Table 2:** Molar concentrations (μM) for the antimicrobial and hemolytic activities of peptides.

Peptide (μg/mL)	Melittin	Px 3	Px 4	Px 5	Px 6	Px 7	Px 11	Px 13
200	70.3	111.3	90.4	90.2	108.8	80	106.5	118.7
100	35.1	55.6	45.2	45.1	54.4	40	53.2	59.3
50	17.6	27.8	22.6	22.6	27.2	20	26.6	29.7
25	8.8	13.9	11.3	11.3	13.6	10	13.3	14.8
12.5	4.4	7.0	5.6	5.6	6.8	5	6.7	7.4
6.25	2.2	3.5	2.8	2.8	3.4	2.5	3.3	3.7

## Supplementary Materials

The following are available online at https://www.mdpi.com/2075-4450/11/11/776/s1, Table S1: Summary of sequencing and annotation, Table S2: Antimicrobial peptide properties prediction and filtration.
