# Supplementary material for: Deciphering Novel Antimicrobial Peptides from the Transcriptome of Papilio xuthus
Source: Insects. 2020 Nov 10;11(11):776. doi: 10.3390/insects11110776 (PMC7697948; doi:10.3390/insects11110776)
Supplement: Supplementary file 1 [file insects-11-00776-s001.pdf]

insects

Table 1. Summary of sequencing and annotation.

Name	Raw Reads	Bases	Q30%	Clean Reads	Bases
I-Px-1	57,222,736	5,779,496,336	91.85	52,253,998	5,236,845,724
	56,551,032	5,711,654,232			
	59,319,708	5,991,290,508			
	59,713,828	6,031,096,628			
I-Px-2	52,750,266	5,327,776,866			
I-Px-3	59,246,872	5,983,934,072			
N-Px-1					
N-Px-2					
N-Px-3	38,272				
**De Novo Assembly**			92.12	51,909,486	5,203,036,297
Unigenes			91.68	54,103,798	5,426,640,422
Bases	38,654,416		93.48	54,783,736	5,487,460,483
Average length of unigenes	1,010		89.11	45,199,918	4,530,252,216
**Annotation**			92.02	54,258,870	5,434,096,474
**Descriptions**	**No of Hits**	**Percentage**			
BLAST- Swissprot	19,900	52			
No blast					
Gene Ontology	18,372	48			
Interproscan	13,945	36			
	13,842	36			

I-Immunized; N-non-immunized.

Table 2. Antimicrobial peptide properties prediction and filtration.

Propensity	Methods	Descriptions/Parameters	Cutoff	No. of Sequences
	Raw Sequence	Total Given Protein Sequences		28543
	Raw Sequence	Small Proteins/Peptides	≤100	9846
Molecular	EPESTFIND	Proteins contains potential protease cleavage site	FALSE	5146
	AMPA	Antimicrobial Spots	≥1	4361
	Pepstats	Total Peptides	≤100	4361
	Pepstats	Peptide Length	≤50	1653
	Pepstats	Small Proteins which have AMP peptides	≤50	1216
	Pepstats	Charge	>0	3996
	Pepstats	Isoelectric Point(pI)	8 ≤ pI ≤ 12	3212
Aggregation (Invivo)	Tango	AGG	≤ 500	3174
	Tango	Helix	0 ≤ Helix ≤ 25	2499
	Tango	Beta	25 ≤ Beta ≤ 100	795
Aggregation (Invitro)	Aggrescan	Na4vSS	-40 ≤ Na4vSS ≤ 60	3960
Allergen	Allerdictor	Predictions	Non-Allergen	4350
Homologus	Blast	Novel	No Blast Hits	4279
	Blast	Known	Blast Hits	82
AMP	CAMP	Support Vector Machine (SVM) Classifier	AMP	1459
		Random Forest Classifier	AMP	1634
		Artificial Neural Network (ANN) Classifier	AMP	1650
		Discriminant Analysis Classifier	AMP	1550
		Predicted >=3 out of 4 Classifiers	≥3	1339
